# Comparative Genomic Analysis Reveals Preserved Features in Organohalide-Respiring *Sulfurospirillum* Strains

**DOI:** 10.1128/msphere.00931-21

**Published:** 2022-02-23

**Authors:** Yi Yang, Torsten Schubert, Yan Lv, Xiuying Li, Jun Yan

**Affiliations:** a Key Laboratory of Pollution Ecology and Environmental Engineering, Institute of Applied Ecology, Chinese Academy of Sciences, Shenyang, Liaoning, China; b Research Group Anaerobic Microbiology, Institute of Microbiology, Friedrich Schiller University Jena, Jena, Germany; c University of Chinese Academy of Sciences, Beijing, China; University of Iowa

**Keywords:** *Sulfurospirillum*, comparative genomics, evolution, organohalide respiration

## Abstract

*Sulfurospirillum* species strains are frequently detected in various pristine and contaminated environments and participate in carbon, sulfur, nitrogen, and halogen elements cycling. Recently we obtained the complete genome sequences of two newly isolated *Sulfurospirillum* strains, ACS_DCE_ and ACS_TCE_, capable of dechlorinating tetrachloroethene to *cis*-1,2-dichloroethene and trichloroethene under low-pH conditions, but a detailed analysis of these two genomes in reference to other *Sulfurospirillum* genomes for an improved understanding of *Sulfurospirillum* evolution and ecophysiology has not been accomplished. Here, we performed phylogenetic and pangenome analyses with 12 completed *Sulfurospirillum* genomes, including those of strain ACS_TCE_ and strain ACS_DCE_, to unravel the evolutionary and metabolic potentials in the genus *Sulfurospirillum*. Based on 16S rRNA gene and whole-genome phylogenies, strains ACS_TCE_, ACS_DCE_, and JPD-1 could be clustered into a single species, proposed as “*Candidatus* Sulfurospirillum acididehalogenans.” TimeTree analysis suggested that the organohalide-respiring (OHR) *Sulfurospirillum* might acquire the ability to use chlorinated electron acceptors later than other energy conservation processes. Nevertheless, the ambiguity of the phylogenetic relations among *Sulfurospirillum* strains complicated the interpretation of acquisition and loss of metabolic traits. Interestingly, all OHR *Sulfurospirillum* genomes except the ones of Sulfurospirillum multivorans strains harbor a well-aligned and conserved region comprising the genetic components required for the organohalide respiration chain. Pangenome results further revealed that a total of 34,620 gene products, annotated from the 12 *Sulfurospirillum* genomes, can be classified into 4,118 homolog families and 2,075 singleton families. Various *Sulfurospirillum* species strains have conserved metabolisms as well as individual enzymes and biosynthesis capabilities. For instance, only the OHR *Sulfurospirillum* species strains possess the quinone-dependent pyruvate dehydrogenase (PoxB) gene, and only “*Ca.* Sulfurospirillum acididehalogenans” strains harbor urea transporter and urease genes. The plasmids found in strain ACS_TCE_ and strain ACS_DCE_ feature genes coding for type II toxin-antitoxin systems and transposases and are promising tools for the development of robust gene editing tools for *Sulfurospirillum*.

**IMPORTANCE** Organohalide-respiring bacteria (OHRB) play critical roles in the detoxification of chlorinated pollutants and bioremediation of subsurface environments (e.g., groundwater and sediment) impacted by anthropogenic chlorinated solvents. The majority of known OHRB cannot perform reductive dechlorination below neutral pH, hampering the applications of OHRB for remediating acidified groundwater due to fermentation and reductive dechlorination. Previously we isolated two *Sulfurospirillum* strains, ACS_TCE_ and ACS_DCE_, capable of dechlorinating tetrachloroethene under acidic conditions (e.g., pH 5.5), and obtained the complete genomes of both strains. Notably, two plasmid sequences were identified in the genomes of strain ACS_TCE_ and strain ACS_DCE_ that may be conducive to unraveling the genetic modification mechanisms in the genus *Sulfurospirillum*. Our findings improve the current understanding of *Sulfurospirillum* species strains regarding their biogeographic evolution, genome dynamics, and functional diversity. This study has applied values for the bioremediation of toxic and persistent organohalide pollutants in low-pH environments.

## INTRODUCTION

Members within the genus *Sulfurospirillum* are capable of versatile energy metabolism (e.g., nitrate reduction and organohalide respiration), enabling them to thrive in various pristine and contaminated environments (1–3). Consequently, *Sulfurospirillum* species strains are considered excellent candidates for biotechnological applications such as oil field souring control and bioremediation of sites impacted by chlorinated contaminants (4). For example, some *Sulfurospirillum* strains had demonstrated nitrate reduction to nitrite, which inhibits the growth of sulfate-reducing bacteria and consequently prevents the souring of oil reservoirs (5). A subset of *Sulfurospirillum* strains (e.g., *S. multivorans* strain DSM 12446, *Sulfurospirillum* sp. strain ACS_TCE_, and *Sulfurospirillum* sp. strain ACS_DCE_) can perform organohalide respiration (OHR) with tetrachloroethene (PCE) and trichloroethene (TCE) as electron acceptors, particularly at a pH as low as 5.5, suggesting that they play key roles for the natural attenuation of anthropogenic chlorinated solvents in acidic environments (2, 4, 6–8). In addition, some *Sulfurospirillum* strains are capable of synthesizing the unconventional norpseudo-type cobamide (i.e., vitamin B_12_ derivative), which can be used to explore the impacts of cobamides on the activities of corrinoid-dependent enzymes and microbial community structures (9). In the contaminated environments, *Sulfurospirillum* strains were frequently found to coexist with obligate organohalide respiring bacteria (OHRB) phylotypes such as Dehalococcoides mccartyi and *Dehalobacter*, which might be explained by their ability to provide the required nutrients, such as cobamides and hydrogen, for these dechlorinators (4). The hypothesis that hydrogen produced by *Sulfurospirillum* via anaerobic oxidation of carboxylic acids enabling syntrophic growth with a hydrogenotrophic partner was later demonstrated in the coculture of *S. multivorans* strain DSM 12446 and Methanococcus voltae (10). Recently, the cross-feeding of hydrogen, acetate, and cobamides was reported during the cocultivation of *S. multivorans* strain DSM 12446 and Dehalococcoides mccartyi strain 195 (11). Based on these observations, *Sulfurospirillum* strains are considered suitable partner populations to explore OHR-related microbial ecology (9–11). Great efforts have been made to investigate the environmental distribution of the genus *Sulfurospirillum*; however, the physiological traits, ecological relevance, and evolutionary history of *Sulfurospirillum* strains are not well understood.

Due to the lack of efficient genetic manipulation systems and difficulties in studying OHR *Sulfurospirillum* using classical biochemical techniques, bioinformatic analysis based on metagenome-assembled genomes (MAGs) and complete genomes are alternative methods to investigate the physiology and metabolic potentials of geographically distributed *Sulfurospirillum* (12). For instance, a genome-wide comparison of *Sulfurospirillum* strains indicated that the non-OHR Sulfurospirillum barnesii strain SES-3, which was isolated from anoxic sediment contaminated with arsenate and selenate, uniquely harbors a rare hybrid gene cluster encoding polyketide synthases (PKS) and nonribosomal peptide synthetases (NRPS). Based on this genome-to-metabolite approach, the first anoxically biosynthesized NRPS-PKS-derived natural product, barnesin A, was identified by omics-guided isolation and total synthesis (13). Additionally, Goris et al. conducted a thorough comparative genomic analysis of *S. multivorans* strain DSM 12446 with closely related non-OHR *S. deleyianum* strain DSM 6946 and *S. barnesii* strain SES-3, which revealed the presence of an ∼50-kbp region containing genes required for OHR and cobamide cofactor biosynthesis and horizontally acquired genes, enabling the catabolic flexibility in *S. multivorans* strain DSM 12446 (14). The genome of *S. cavolei* strain MES, assembled from the metagenomic sequences of an electrosynthetic microbiome, was compared with other 10 complete or draft *Sulfurospirillum* genomes featuring conserved and divergent physiologies and metabolisms. This pangenome analysis revealed a total of 6,264 homolog families, including 1,082 homolog families shared among all 11 *Sulfurospirillum* genomes (i.e., core clusters), 1,991 homolog families shared among part of 11 genomes (i.e., accessory clusters), and 3,191 singleton families (i.e., unique clusters), indicating the commonalities in general functions as well as ecological pressures induced acquisition of unique gene sets (15). Combined with transcriptomics data, a recent *Sulfurospirillum* comparative genomics study on the region encoding OHR genetic components identified a two-component regulator that is responsible for PCE-induced gene expression in OHR *Sulfurospirillum* strains (16).

To date, at least 47 complete or draft *Sulfurospirillum* genomes are publicly available (www.ncbi.nlm.nih.gov/genome). Two novel *Sulfurospirillum* organisms, strain SL2-1 and strain SL2-2, which performed PCE-to-TCE and PCE-to-*cis*-1,2-dichloroethene (*c*DCE) dechlorination, respectively, were recently enriched from a PCE-dechlorinating consortium maintained for 10 years (8). The assembled genomes of strain SL2-1 and strain SL2-2 are highly identical, representing a new *Sulfurospirillum* species proposed as “*Candidatus* Sulfurospirillum diekertiae” (8). The increased numbers of *Sulfurospirillum* genomes require an updated genomic examination and offer the opportunity for comprehensive comparison of OHR and non-OHR *Sulfurospirillum*. Such efforts are promising to provide new insights into the physiological traits, metabolic potentials, and evolution characteristics in the genus *Sulfurospirillum*.

In this study, we performed comparative pangenome analysis on 12 complete *Sulfurospirillum* genomes, including those of two new *Sulfurospirillum* isolates, strain ACS_DCE_ and strain ACS_TCE_, capable of dechlorinating PCE to TCE and *c*DCE, respectively, under low-pH conditions (e.g., pH 5.5). We found that a well-aligned and conserved region comprising the genetic components required for the organohalide respiration chain is present in all OHR *Sulfurospirillum* genomes except the ones in *S. multivorans* strains. Genomic differences between non-OHR and OHR *Sulfurospirillum* strains as well as variations among OHR *Sulfurospirillum* strains were observed and discussed. Findings of this study will advance our understanding of members of the genus *Sulfurospirillum* regarding their evolutionary traits, genome dynamics, and functional diversity.

## RESULTS AND DISCUSSION

### Proposition of “*Candidatus* Sulfurospirillum acididehalogenans” as a new *Sulfurospirillum* species.

**FIG 1:**
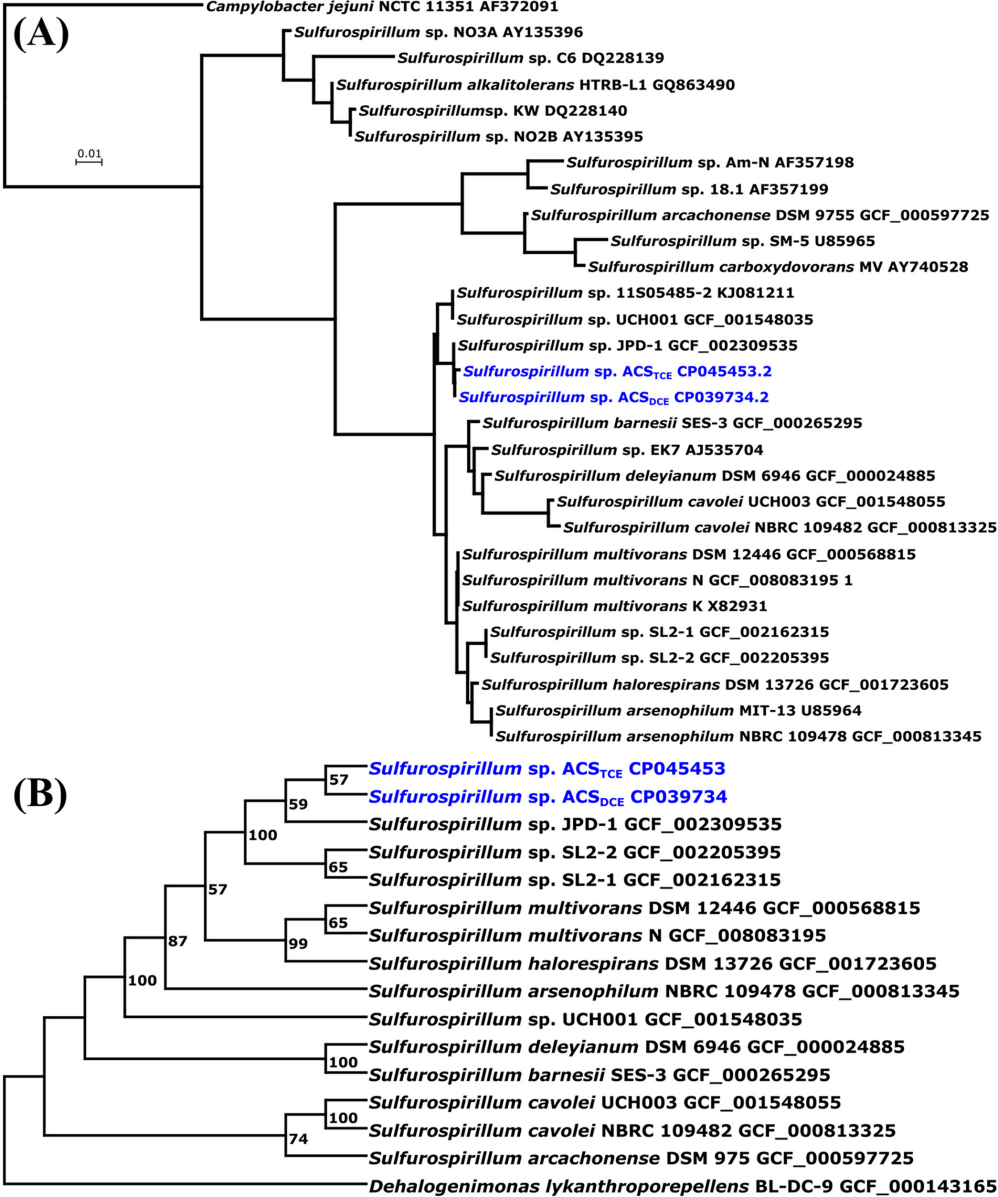
(A) 16S rRNA gene phylogenetic tree of *Sulfurospirillum* species strains. (B) The genome-based phylogenetic tree was inferred with FastME 2.1.6.1 from GBDP distances calculated from genome sequences. The branch lengths are scaled in terms of GBDP distance formula d5. The numbers above branches in panel B are GBDP pseudobootstrap support values of >60% from 100 replications, with an average branch support of 81.7%. Strain ACS_TCE_ and strain ACS_DCE_ are colored blue.

Multiple tools (e.g., JSpeciesWS, TYGS, and GTDB) and analyses (e.g., 16S rRNA genes and whole-genome sequences) were performed to reveal the phylogenetic placement of strain ACS_DCE_ and strain ACS_TCE_ in reference to other *Sulfurospirillum* species strains. Pairwise comparison of 16S rRNA gene sequences demonstrated that strains ACS_DCE_, ACS_TCE_, and JPD-1, which share 99.8 to 99.9% identities to each other, are clustered into a distinct subclade with 90.9% to 98.9% identities to other *Sulfurospirillum* species strains (Fig. 1A). Phylogenetic inference with complete genome sequences further demonstrated that strains ACS_DCE_, ACS_TCE_, and JPD-1 can be placed into a single cluster with “*Candidatus* Sulfurospirillum diekertiae” strain SL2-1 and strain SL2-2 as the closest relatives (Fig. 1B; see also Fig. S2 in the supplemental material). Pairwise comparison of genome sequences performed with TYGS found that strain ACS_DCE_ and strain ACS_TCE_ shared 99.3 to 99.7% dDDH (digital DNA-DNA hybridization) based on three different GBDP formulas (Table S2); by comparison, strain JPD-1 shared 69.3% and 84.7% dDDH with strain ACS_DCE_ and 69.3% and 83.4% dDDH with strain ACS_TCE_ (Table S2). ANIm, ANIb, and orthoANI analyses by JSpeciesWS and orthoANI demonstrated that the calculated ANI values for each pair of strains ACS_TCE_, ACE_DCE_, and JPD-1 were above the 95% threshold for species delineation (Tables S3 and S4, Fig. S3). Based on these results, we proposed to unify strains ACS_DCE_, ACS_TCE_, and JPD-1 into a new *Sulfurospirillum* species, designated “*Candidatus* Sulfurospirillum acididehalogenans.”

10.1128/msphere.00931-21.3 FIG S2 Phylogenetic tree of *Sulfurospirillum* species based on GTDB-tk classification and GTDB analysis. Strains JPD-1, ACS_TCE_, and ACS_DCE_ were classified into a single species, designated “*Sulfurospirillum* sp002309535” (colored in blue), with strain JPD-1 as the type strain, while strains SL2-1 and SL2-2 were classified into another species, designated “*Sulfurospirillum* sp002162315” (i.e., “*Sulfurospirillum diekertiae*”). Download FIG S2, TIF file, 0.3 MB. Copyright © 2022 Yang et al. 2022 Yang et al. https://creativecommons.org/licenses/by/4.0/ This content is distributed under the terms of the Creative Commons Attribution 4.0 International license.

10.1128/msphere.00931-21.4 FIG S3 Classification and ANI values for selected *Sulfurospirillum* strains performed by OrthoANI software. Download FIG S3, TIF file, 0.3 MB. Copyright © 2022 Yang et al. 2022 Yang et al. https://creativecommons.org/licenses/by/4.0/ This content is distributed under the terms of the Creative Commons Attribution 4.0 International license.

### OHR *Sulfurospirillum* species diverged from non-OHR *Sulfurospirillum* recently.

**FIG 2:**
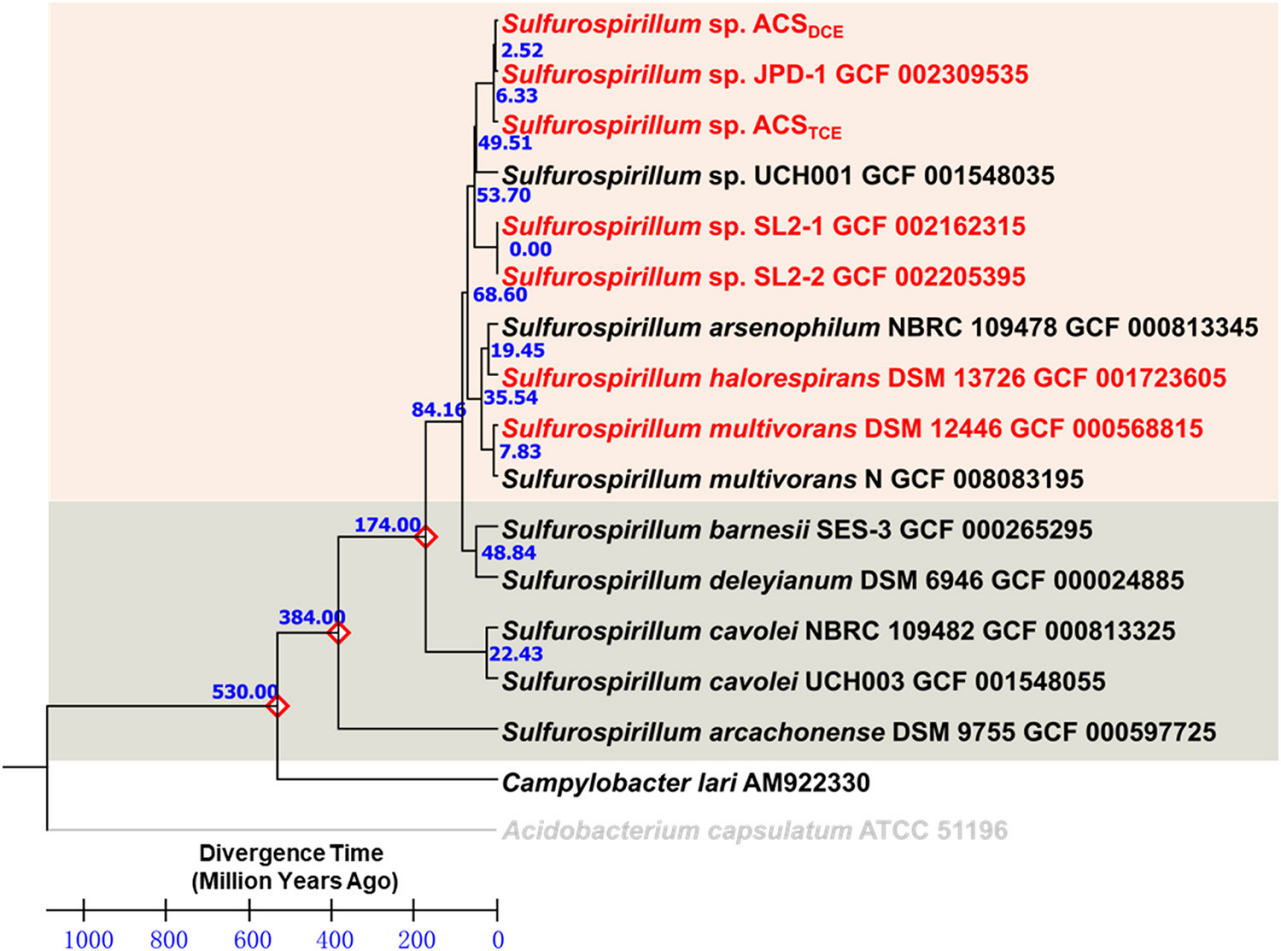
Phylogeny and molecular clock of selected 16 rRNA genes from *Sulfurospirillum* species strains and Campylobacter lari from *Proteobacteria*. Acidobacterium capsulatum ATCC 51196 was chosen as the outgroup. Branch lengths represent the divergence times (MYA) approximated by the RelTime method using Mega X. The OHR and non-OHR clusters of *Sulfurospirillum* species strains were colored in light orange and gray, respectively.

Genetic processes (e.g., mutation, horizontal gene transfer) and extreme geological events (e.g., great oxygenation event, neoproterozoic oxygenation event) can affect the long-term evolution of microorganisms; however, our understanding of microbial evolution is limited by the lack of geological and biological evidence (e.g., fossils, genetics) through the geological time scale. We applied the TimeTree analysis with the RelTime method to evaluate the evolution time frame of several newly sequenced *Sulfurospirillum* species strains by following the well-established protocols (17, 18). The TimeTree analysis suggested that the ancestor of the new species “*Candidatus* Sulfurospirillum acididehalogenans” emerge approximately between the Eocene and Miocene (e.g., between 49.41 and 6.33 million years ago [MYA]) (Fig. 2), which was much later than the estimated appearance time (e.g., the Neoproterozoic era, 1,000 to 541 MYA) of obligate organohalide respiring *Dehalococcodia* (18). The TimeTree analysis is based on 16S rRNA gene sequences and intended to infer the divergence times of strains within the genus *Sulfurospirillum*. Such an approach can only provide a rough estimate; however, this effort reflected our interests in unresolved questions, including (i) how does the organohalide respiration metabolism evolve, (ii) when does this energy conservation emerge in geoscale time, and (iii) when were the organohalide-respiratory genes transferred into microorganisms of different genera (e.g., *Sulfurospirillum*). To the best of our knowledge, no solid evidence is available to predict or support when an insertion event (e.g., the organohalide respiration region) in the *Sulfurospirillum* genome occurs, since dating the hypothetical insertion event is still difficult. Nevertheless, the dechlorinating *Sulfurospirillum* species strains could not obtain organohalide respiration genes before their own existence; therefore, the insertion event or organohalide respiration genes horizontally transferred occurred roughly at the same time or later than the divergence time of *Sulfurospirillum* species strains.

Furthermore, a previous genomic comparison study based on the genome sequence of Sulfurospirillum multivorans discovered a 50-kbp organohalide-respiring region (14), which was also identified in the genome of another species, Sulfurospirillum halorespirans (6), indicating the ability to respire halogenated organic compounds was horizontally acquired by some ancestors of the genus *Sulfurospirillum* recently. Ancestors of the OHR *Sulfurospirillum* strains may diverge from the non-OHR *Sulfurospirillum* strains via horizontal acquisition of the genetic components required for organohalide respiration chain and *de novo* cobamide biosynthesis, and OHR *Sulfurospirillum* strains were subsequently distributed to different niches on the planet Earth, which could be inferred by the observation that different OHR *Sulfurospirillum* strains with conserved arrangements of the gene cluster responsible for OHR were isolated from geographically distinct origins. Nonetheless, the non-OHR *Sulfurospirillum* sp. strain UCH001, which was isolated from chlorinated ethene-contaminated groundwater in Japan but could not dechlorinate chlorinated ethenes (19), was closely related to the OHR *Sulfurospirillum* strains. Such an inconsistency may be due to the loss of gene clusters responsible for organohalide respiration and requires further investigation.

### Shared features in the chromosomes of strain ACS_DCE_ and strain ACS_TCE_.

The chromosome of strain ACS_TCE_ has 2,998 features, including 2,853 coding genes, 3 CRISPR (clustered regularly interspaced short palindromic repeats) arrays, 26 CRISPR repeats, 23 CRISPR spacers, and 50 noncoding RNAs. Only 1,338 coding sequences could be assigned with a SEED (https://pubseed.theseed.org) annotation ontology across 910 distinct SEED functions. By comparison, the chromosome of strain ACS_DCE_ harbors 2,993 features, including 2,852 coding genes, 3 CRISPR arrays, 26 CRISPR repeats, 23 CRISPR spacers, and 55 noncoding RNAs. Only a total of 1,351 coding sequences could be assigned with a SEED annotation ontology across 911 distinct SEED functions. Functioning-based metabolic reconstruction comparison between strain ACS_DCE_ and strain ACS_TCE_ suggested that both strains share a total of 956 functioning roles defined within the SEED subsystems (e.g., biotin biosynthesis, cobamide synthesis, coenzyme A biosynthesis, and heme and siroheme biosynthesis) (Table S5). BLAST of the annotated coding sequences showed that the homologs of 70 genes in the chromosome of strain ACS_DCE_ were not found in the chromosome of strain ACS_TCE_, including those genes encoding hydroxylamine reductase, arginyl-tRNA protein transferase, DNA helicase restriction/modification system component YeeBC, and 50 hypothetical genes with unknown functions (Table S6). Likewise, 23 coding genes in the chromosome of strain ACS_TCE_ were not matched in the chromosome of strain ACS_DCE_, such as genes encoding cytochrome *c*_552_ precursor, anaerobic C_4_-dicarboxylate transporter DcuA, heterodisulfide reductase subunit B-like protein/putative succinate dehydrogenase subunit, and other 10 hypothetical proteins (Table S7). Genes encoding tetrathionate reductase, nitrate reductase, formate dehydrogenase, and [NiFe] hydrogenase were present in both chromosomes of strains ACS_TCE_ and strain ACS_DCE_. However, no genes related to nitrite ammonification were identified, suggesting that they can reduce nitrate as an electron acceptor and produce nitrite only. In pure cultures, strain ACS_TCE_ and strain ACS_DCE_ could transform up to 60% of an initial 5 mM nitrate to nitrite (Fig. S4 and S5). Whether the accumulation of nitrite from nitrate transformation inhibits the activities of *Sulfurospirillum* and whether strains ACS_DCE_ and ACS_TCE_ can transform nitrite to ammonia require further investigation.

10.1128/msphere.00931-21.5 FIG S4 Nitrate reduction by *Sulfurospirillum* strain ACS_TCE_ with different starting nitrate concentrations (i.e., 1, 5, 10, and 20 mM). Download FIG S4, TIF file, 0.2 MB. Copyright © 2022 Yang et al. 2022 Yang et al. https://creativecommons.org/licenses/by/4.0/ This content is distributed under the terms of the Creative Commons Attribution 4.0 International license.

10.1128/msphere.00931-21.6 FIG S5 Nitrate reduction by *Sulfurospirillum* strain ACS_DCE_ with different starting nitrate concentrations (i.e., 1, 5, 10, and 20 mM). Download FIG S5, TIF file, 0.2 MB. Copyright © 2022 Yang et al. 2022 Yang et al. https://creativecommons.org/licenses/by/4.0/ This content is distributed under the terms of the Creative Commons Attribution 4.0 International license.

### Potential roles of plasmids in strain ACS_DCE_ and strain ACS_TCE_.

Except for strain ACS_TCE_ and strain ACS_DCE_, plasmids are not found in *Sulfurospirillum* species strains, presumably due to the absence of plasmid or difficulty in faithful assembly and identification of plasmid solely using Illumina short reads (20). In contrast, circular plasmids with sizes of 38,046 bp and 39,868 bp were assembled from the genome sequencing data of strain ACS_TCE_ and strain ACS_DCE_, respectively, using PacBio long reads and Illumina short reads (21, 22). Plasmid sequence alignment by MAUVE demonstrated that the synteny of the two plasmid sequences was not conserved, and only two regions with a total size of 9.5 kbp could be mapped between the two plasmid sequences. Plasmids of strain ACS_TCE_ and strain ACS_DCE_ carried 94 and 57 coding sequences, of which only 19 and 20 encoded hypothetic proteins, respectively. By comparison, the eggNOG-mapper pipeline found that 37 coding sequences of strain ACS_TCE_ plasmid and 32 of strain ACS_DCE_ plasmid (Table S8 and S9) matched with orthologous groups in the eggNOG database. While most of the orthologous genes related to the coding sequences in the plasmids of strain ACS_TCE_ and strain ACS_DCE_ were found in Proteobacteria, a few coding sequences (e.g., *parA* and *mcrB*) present in these two plasmids were closely related to those found in *Clostridia* of Firmicutes and Bacteroidetes (Tables S8 and S9). Therefore, we hypothesized that these two plasmids originate from Proteobacteria with additional coding sequences horizontally acquired from other phyla (e.g., Firmicutes). Two prophage-related regions with lengths of 20,367 bp and 26,549 bp were found in the plasmids of strain ACS_TCE_ and strain ACS_DCE_, respectively. In addition, the plasmid and chromosomal sequences of strain ACS_TCE_ shared an identical repeat region with an approximate size of 1 kbp and was in strain ACS_DCE_, indicating the exchange of genetic materials between the plasmid and the chromosome in both strains. Sequences encoding components (e.g., a toxic protein and its cognate antitoxin protein) of various type II toxin-antitoxin (TA) systems (e.g., HicA-HicB, YafQ-DinJ, and RelE-RelB) are both present in the plasmids of the strain ACS_TCE_ and strain ACS_DCE_. Type II TA systems have been proposed to play roles in genome stabilization, abortive phage infection, stress modulation, and antibiotic persistence (23); however, how such a system is related to the survival of strain ACS_TCE_ and strain ACS_DCE_ is unknown. Four and three genes coding for transposases were annotated from the plasmids of strain ACS_TCE_ and strain ACS_DCE_, respectively. A phylogenetic analysis on transposases annotated from *Sulfurospirillum* genomes indicated that five transposases from the plasmid sequences were clustered within the IS30/IS982 family. One IS21 family transposase on the plasmid of strain ACS_DCE_ was clustered with the transposases annotated on the chromosomes of strain ACS_DCE_ and strain ACS_TCE_ (Fig. S6). Restriction sites for restriction endonucleases such as BamHI, BglII, EcoRI, PvuI, and SalI were identified in both plasmids, providing retrospective *in silico* evidence of the movement of genomic sequences between the plasmid and chromosome in strain ACS_TCE_ and strain ACS_DCE_. Overall, these results improve our understanding of the genomic characteristics of *Sulfurospirillum* species strains and are promising for the development of molecular tools for editing *Sulfurospirillum* genomes.

10.1128/msphere.00931-21.7 FIG S6 Phylogenetic analysis of transposase sequences encoded on the plasmids of strain ACS_TCE_ and strain ACS_DCE_ (colored in blue) and transposases from the chromosomes of select *Sulfurospirillum* species strains. Transposases were verified using ISFinder server and database (https://www-is.biotoul.fr/about.php) with two representative families labeled with red and green colors. The scale bar represents substitution per site. Download FIG S6, TIF file, 0.5 MB. Copyright © 2022 Yang et al. 2022 Yang et al. https://creativecommons.org/licenses/by/4.0/ This content is distributed under the terms of the Creative Commons Attribution 4.0 International license.

### Pangenome analysis revealed conserved and differed features in OHR *Sulfurospirillum*.

**FIG 3:**
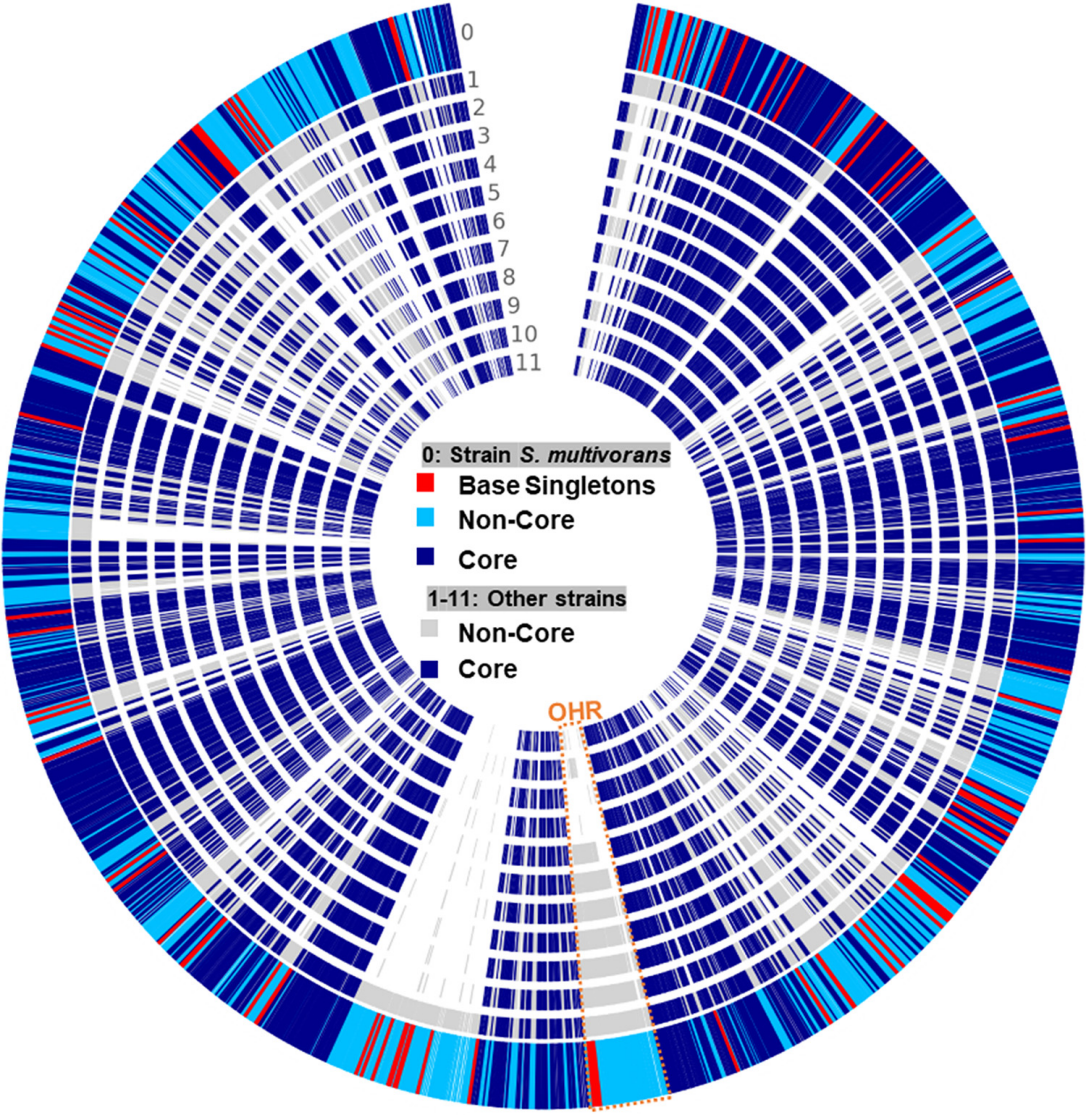
Circle view of pangenome with *S. multivorans* strain DSM 12446 as the base genome. The order of the genes in the rest of the pangenome is aligned to the position of its ortholog in the base genome. Genomes 1 to 11 are the following: 1, strain N; 2, *S. halorespirans*; 3, strain SL2-1; 4, strain SL2-2; 5, strain JPD-1; 6, strain ACS_DCE_; 7, strain ACS_TCE_; 8, strain UCH003; 9, strain UCH001; 10, strain SES-3; 11, *S. deleyianum*. The OHR colored in orange stands for the organohalide respiration region.

A total of 12 complete *Sulfurospirillum* genomes were selected for genomic comparison analysis to unravel the core functions and core protein families using the OrthoMCL tool, including seven experimentally verified OHR *Sulfurospirillum* genomes (i.e., strains ACS_DCE_, ACS_TCE_, JPD-1, SL2-1, SL2-2, *S. multivorans* DSM 12446, and *S. halorespirans* DSM 13726) and four non-OHR *Sulfurospirillum* genomes (i.e., strains UCH001, UCH003, *S. deleyianum* DSM 6946, and *S. barnesii* SES-3). *S. multivorans* strain N was demonstrated to be incapable of dechlorinating chlorinated ethenes despite its genome harbors two homologous reductive dehalogenase genes. For pangenome analysis, we grouped strain N with other OHR *Sulfurospirillum* species strains, since strain N may have lost its OHR capability recently by a transposition event (16). Generally, all examined *Sulfurospirillum* species strains shared a variety of conserved sequence regions (Fig. 3). Furthermore, whole-sequence alignment of all OHR *Sulfurospirillum* species strains by Mauve demonstrated that all of them except *S. multivorans* strain DSM 12446 and *S. multivorans* strain N have a larger well-aligned and conserved regions (i.e., 330 kbp) containing a previously proposed 50-kb OHR region (Fig. S7). A 148-kbp block that was not found in other OHR *Sulfurospirillum* genomes was inserted into the end of the OHR region only in the genomes of *S. multivorans* strain DSM 12446 and *S. multivorans* strain N. The OHR regions of eight OHR strains were estimated to be between 63 kbp (e.g., *S. halorespirans*) and 74 kbp (e.g., *S. multivorans*). A total of 34,620 coding sequences (i.e., 32,545 homolog gene sequences and 2,075 singleton gene sequences), annotated from 12 *Sulfurospirillum* genomes, was classified into 6,193 families (i.e., 4,118 homolog families and 2,075 singleton families). The average numbers of gene sequences, genes in homologs, genes in singletons, and homolog families for the total of 12 *Sulfurospirillum* genomes were 2,885, 2,712, 173, and 2,625, respectively. By comparison, the average numbers of gene sequences, genes in homologs, and homolog families, but not genes in singletons, found in the eight OHR *Sulfurospirillum* genomes were larger than those of the four non-OHR *Sulfurospirillum* genomes, probably because all OHR *Sulfurospirillum* species strains have larger genome sizes than those of non-OHR *Sulfurospirillum* species strains by horizontally acquiring the genomic regions responsible for OHR (Table S1). The shared homolog families for each pair of *Sulfurospirillum* genomes were summarized in Table 1.

**TABLE 1:** Pairwise comparisons of 12 *Sulfurospirillum* genomes for the shared homolog families

Homolog family	G1	G2	G3	G4	G5	G6	G7	G8	G9	G10	G11	G12
G1, *S. deleyianum*	**2,085**	1,847	1,849	1,747	1,776	1,781	1,794	1,794	1,773	1,746	1,854	1,845
G2, strain N	1,847	**3,146**	2,050	2,089	2,133	2,248	2,309	2,308	2,227	2,187	2,534	3,140
G3, strain SES-3	1,849	2,050	**2,255**	1,812	1,847	1,862	1,906	1,903	1,827	1,813	2,001	2,050
G4, strain UCH001	1,747	2,089	1,812	**2,307**	2,030	2,062	2,104	2,102	2,070	2,052	2,113	2,088
G5, strain UCH003	1,776	2,133	1,847	2,030	**2,389**	2,096	2,109	2,107	2,074	2,057	2,154	2,133
G6, strain JPD-1	1,781	2,248	1,862	2,062	2,096	**2,632**	2,485	2,484	2,427	2,376	2,303	2,243
G7, strain SL2-1	1,794	2,309	1,906	2,104	2,109	2,485	**2,797**	2,779	2,394	2,351	2,347	2,306
G8, strain SL2-2	1,794	2,308	1,903	2,102	2,107	2,484	2,779	**2,791**	2,391	2,351	2,343	2,305
G9, strain ACS_DCE_	1,773	2,227	1,827	2,070	2,074	2,427	2,394	2,391	**2,628**	2,565	2,253	2,224
G10, strain ACS_TCE_	1,746	2,187	1,813	2,052	2,057	2,376	2,351	2,351	2,565	**2,578**	2,214	2,187
G11, *S. halorespirans*	1,854	2,534	2,001	2,113	2,154	2,303	2,347	2,343	2,253	2,214	**2,750**	2,531
G12, *S. multivorans*	1,845	3,140	2,050	2,088	2,133	2,243	2,306	2,305	2,224	2,187	2,531	**3,141**

10.1128/msphere.00931-21.8 FIG S7 Complete alignment of genome sequences of OHR *Sulfurospirillum* species strains as computed by Mauve. Download FIG S7, TIF file, 0.9 MB. Copyright © 2022 Yang et al. 2022 Yang et al. https://creativecommons.org/licenses/by/4.0/ This content is distributed under the terms of the Creative Commons Attribution 4.0 International license.

**FIG 4:**
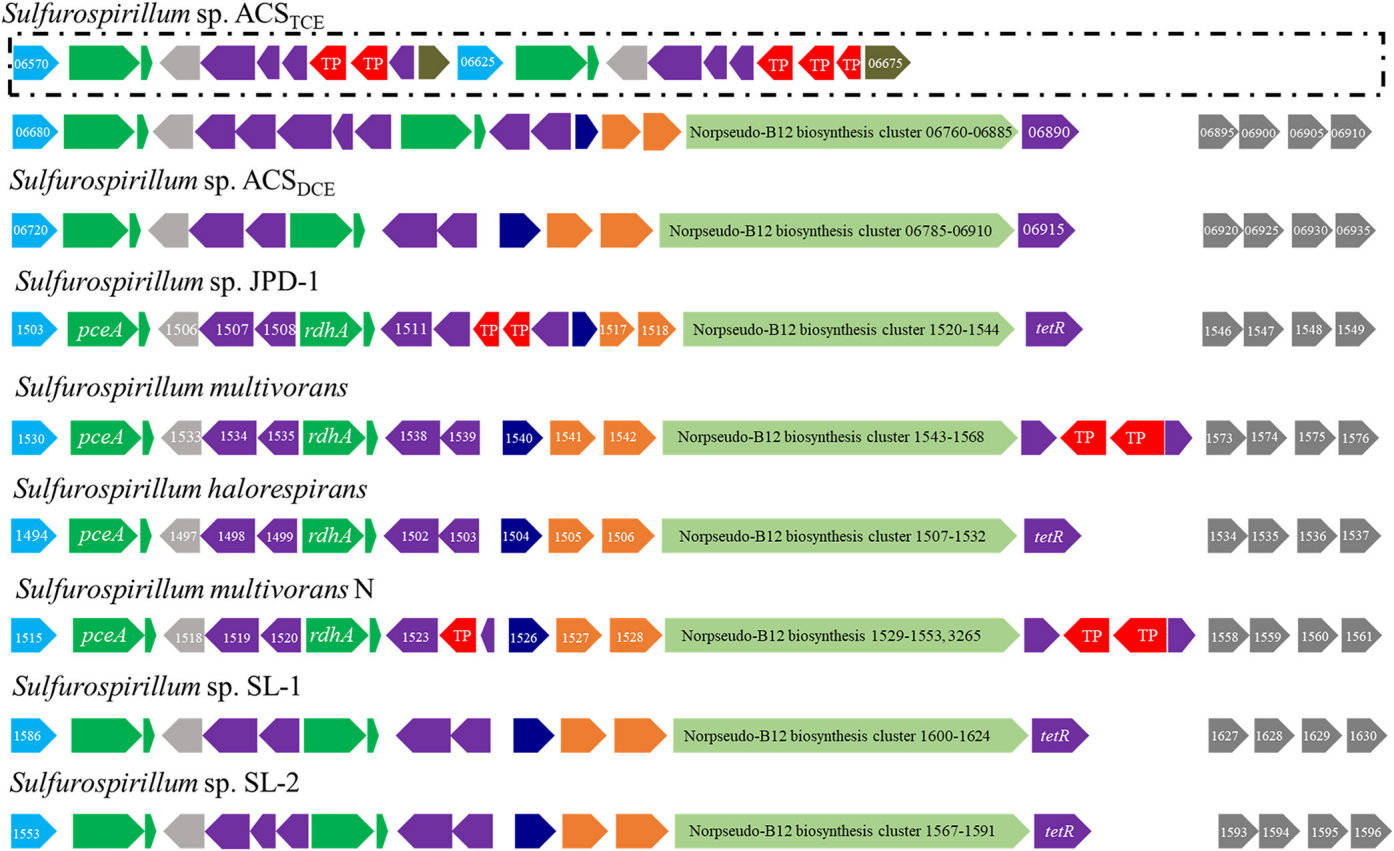
Illustration of the preserved OHR-related gene regions in the OHR *Sulfurospirillum* species strains. These regions encode carboxymuconolactone decarboxylase family protein (light blue), followed by the reductive dehalogenase with associated putative membrane anchor protein (green), iron-sulfur cluster assembly scaffold protein (light gray), sensor histidine kinase and response regulator transcription factor (purple), DUF4405 domain-containing protein (dark blue), components of a putative quinol dehydrogenase (orange), norcobamde biosynthesis clusters (light green), and transposase (red). The OHR region of strain ACS_TCE_ possesses two additional reductive dehalogenase gene clusters presented in the dashed box.

A total of 63 homolog families was only found in the genomes of OHR *Sulfurospirillum* species strains, including homologous genes encoding reductive dehalogenase, quinone-dependent pyruvate dehydrogenase (i.e., PoxB), NADPH-dependent FMN reductase, short-chain dehydrogenases/reductase, transcriptional regulator AraC family, Ser-tRNA(Ala) deacylase/Gly-tRNA(Ala) deacylase, phosphinothricin N-acetyltransferase, proteins associated with cobamide transport and biosynthesis (e.g., vitamin B_12_-ABC transporter permease component BtuC, B_12_-binding component BtuF, uroporphyrinogen-III methyltransferase/uroporphyrinogen-III synthase, and cobalt-precorrin-6A reductase), and a cluster related to propanoate metabolism (e.g., acetyl-coenzyme A synthetase, methylisocitrate lyase, 2-methylcitrate synthase, and 2-methylcitrate dehydratase). The previously reported 50-kbp gene region (e.g., 54 to 61 coding sequences starting from the gene representing carboxymuconolactone decarboxylase family protein) containing reductive dehalogenase genes and the gene cluster coding for (nor)cobamide biosynthesis from uroporphyrinogen III (14, 24) were conserved in all eight OHR *Sulfurospirillum* genomes (Fig. 4) but not present in the non-OHR *Sulfurospirillum* genomes. The only exception is that *S. barnesii* strain SES-3 of the non-OHR *Sulfurospirillum* group has several genes encoding *de novo* cobamide biosynthesis (Fig. 3) (14). All eight OHR *Sulfurospirillum* species strains and the non-OHR *S. cavolei* strain UCH003 possess the complete gene set for nitrogen fixation. All examined *Sulfurospirillum* species strains except *S. barnesii* strain SES-3 and *S. deleyianum* strain DSM 6946 possess the complete gene set for tetrathionate reductase.

**FIG 5:**
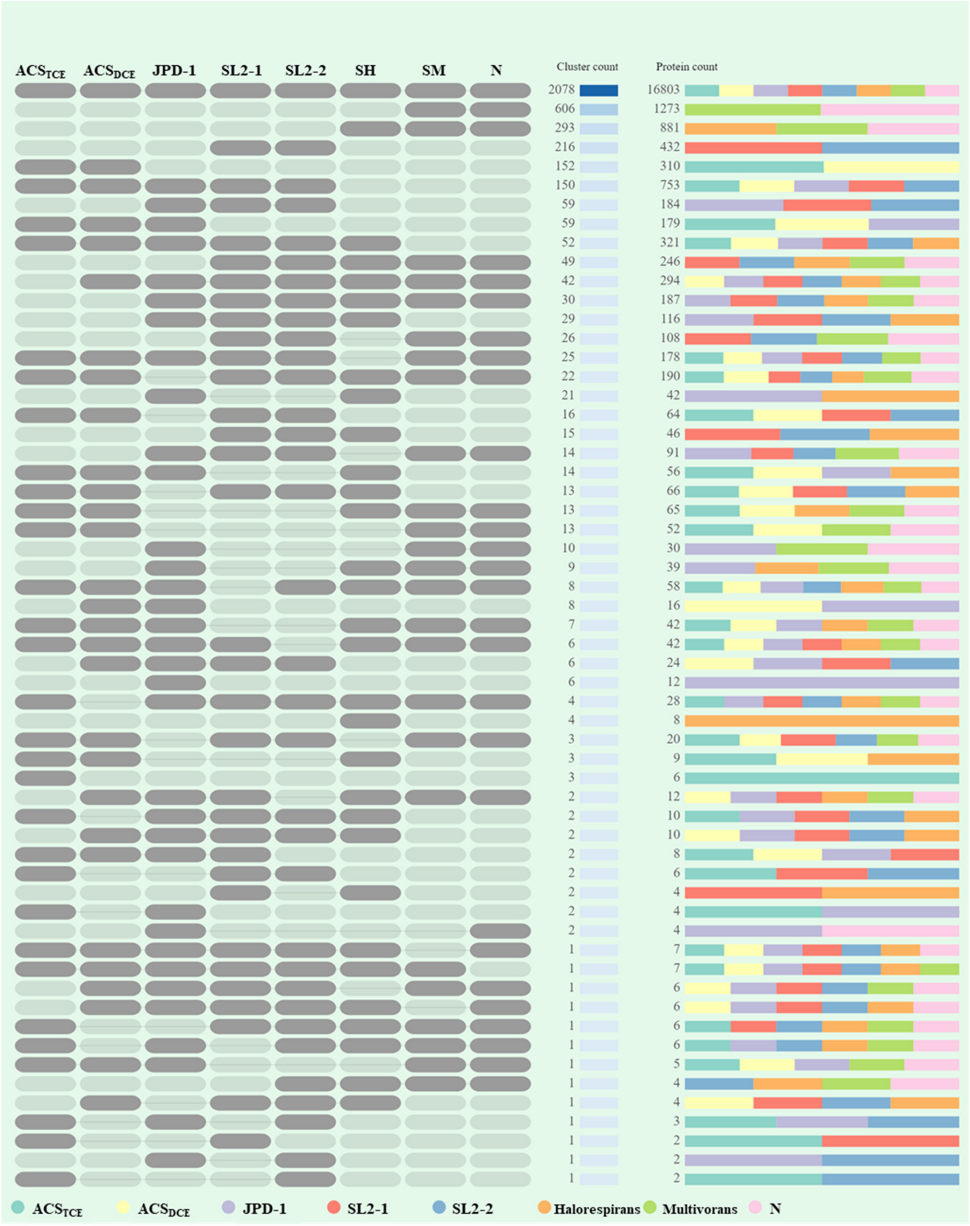
Orthologous gene clusters across eight OHR *Sulfurospirillum* strains. The filled and gray blocks indicate the presence and absence of orthologous gene clusters in each genome. SH, SM, and N stand for *S. halorespirans*, *S. multivorans*, and strain N, respectively.

The eight OHR *Sulfurospirillum* genomes contain a total of 4,113 gene clusters (Fig. 5). Genomes of “*Candidatus* Sulfurospirillum acididehalogenans” strains ACS_TCE_, ACS_DCE_, and JPD-1 shared 59 clusters consisting of 179 coding sequences (Fig. 5). Notably, “*Candidatus* Sulfurospirillum acididehalogenans” strains ACS_DCE_, ACS_TCE_, and JPD-1 have a 14-genes cluster encoding urea transporter UrtABCDEFG, urease, two-component sensor histidine kinase, and a hybrid sensor histidine kinase/response regulator. The presence of urease is hypothesized to allow strains ACS_DCE_, ACS_TCE_, and JPD-1 to tolerate low-pH conditions by yielding ammonia to neutralize protons (25); however, *S. multivorans* also can grow and dechlorinate PCE under acidic conditions (i.e., pH 5.5) and does not possess the urease gene cluster. “*Candidatus* Sulfurospirillum acididehalogenans” and *S. multivorans* probably could possess additional acid tolerance mechanisms, such as involvement of F_0_F_1_-ATPase in pH homeostasis, amino acid-dependent decarboxylase/antiporter systems, and deiminase and deaminase systems. Genes encoding F_0_F_1_-ATP synthase and various decarboxylase (e.g., arginine decarboxylase, aspartate decarboxylase) and agmatine deiminase family proteins were present in the genomes of “*Candidatus* Sulfurospirillum acididehalogenans” and *S. multivorans*. Comparatively, a total of 246 coding sequences from all OHR *Sulfurospirillum* species strains except “*Candidatus* Sulfurospirillum acididehalogenans” strains ACS_DCE_, ACS_TCE_, and JPD-1 were classified into 49 clusters, including genes for the l-proline glycine betaine ABC transport system (i.e., ProVWX), arsenite oxidase, 2-oxoglutarate/malate translocator, ferric iron ABC transporter, respiratory arsenate reductase Mo binding, and FeS subunits ArrAB. Strain ACS_TCE_ and strain ACS_DCE_ shared 152 orthologous gene clusters containing 310 coding sequences, most of which code for CRISPR-associated proteins, transposases, and mobile element proteins. Two adjacent genes annotated as carbon monoxide dehydrogenases, CooS, and carbon monoxide dehydrogenase accessory protein, CooC, were present in the genomes of strain ACS_DCE_ and strain ACS_TCE_ but not in other *Sulfurospirillum* genomes. By comparison, all the OHR *Sulfurospirillum* species strains, except strain ACS_DCE_ and strain ACS_TCE_, harbor genes encoding polysulfide reductase NrfD, potassium-transporting ATPase, nitrous oxide reductase NosZ, and nitrous oxide reductase maturation protein NosL.

### Differences between PCE-to-*c*DCE and PCE-to-TCE dechlorinating reductive dehalogenases.

**FIG 6:**
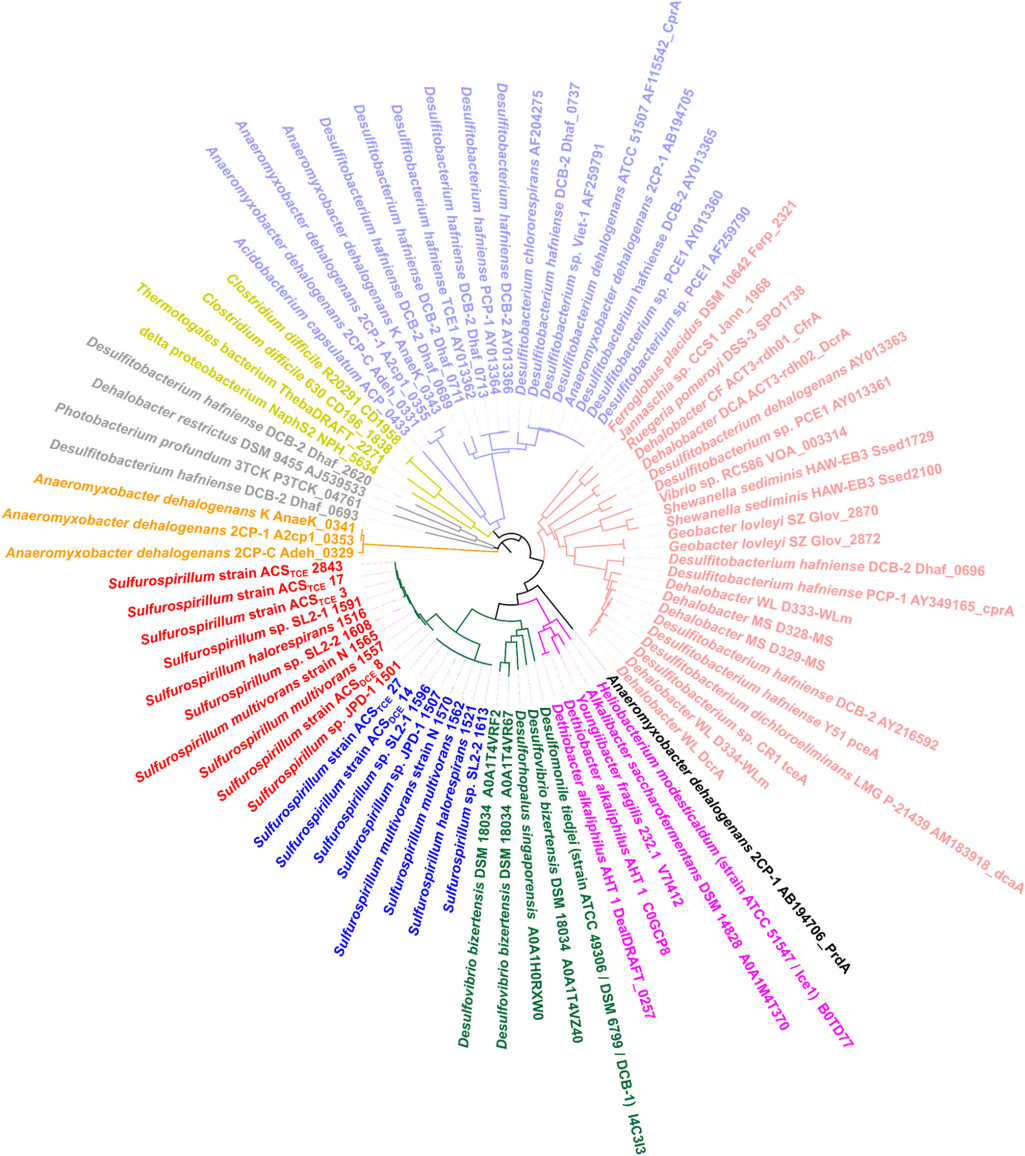
Phylogenetic tree of RdhAs from OHR *Sulfurospirillum* and select OHRB. The putative RdhAs were retrieved from the OGs database (29). Clusters I and II of *Sulfurospirillum* RdhAs were colored in blue and red, respectively.

All examined OHR *Sulfurospirillum* genomes possess two copies of reductive dehalogenase homologous (RdhA) genes, except that four RdhA copies were found in the genome of strain ACS_TCE_ (Fig. 4). Each of these RdhA genes is adjacent to a gene with a size of 150 to 225 bp encoding reductive dehalogenase membrane anchor protein RdhB. Phylogenetic analysis indicated that all 18 RdhAs encoded by the genomes of known *Sulfurospirillum* species strains can be grouped into two distinct clusters, with several RdhAs belonging to the genera *Desulfovibrio*, *Desulfomonile*, and *Desulforhopalus* as the closet relatives (Fig. 6). Seven RdhAs in cluster I (colored in blue in Fig. 6) were 100% identical to each other, but the RdhA from strain ACS_TCE_ shares 99.8% amino acid similarity with the other seven RdhAs in cluster I. The substrate spectrum of the eight putative RdhAs in cluster I remains elusive. By comparison, the three identical RdhAs (ACS_TCE__3, ACS_TCE__17, and ACS_TCE__2843) of strain ACS_TCE_ and the other five putative RdhAs (Shal_1516, JPD-1_1501, SL2-2_1608, ACS_DCE__8 and Smul_N_1565) in cluster II (colored red in Fig. 6) were grouped with the characterized PceA (Smul_1557) of *S. multivorans* strain DSM 12446, indicating that they can dechlorinate PCE. The putative RdhAs of Shal_1516, JPD-1_1501, SL2-2_1608, ACS_DCE__8, and Smul_N_1565 shared 92.1%, 94.0%, 96.2%, 97.2%, and 99.8% similarities, respectively, with PceA of *S. multivorans*. The three identical RdhAs of strain ACS_TCE_ (colored red in Fig. 6) share 97.8% similarity with the RdhA of strain SL2-1 (SL2-1_1591), and these four RdhAs were predicted to be responsible for dechlorinating PCE to TCE based on the fact that strain ACE_TCE_ and strain SL2-1 could only dechlorinate PCE to TCE. Only four critical residue differences (Ser^279^ versus Ala^279^, Gly^286^ versus Cys^286^, Ser^312^ versus Cys^312^, and Pro^320^ versus Ala^320^) were identified among the four putative PCE-to-TCE dechlorinating RdhAs (SL2-1_1591, ACS_TCE__3, ACS_TCE__17, and ACS_TCE__2843) and the other six putative PCE-to-*c*DCE dechlorinating RdhAs (Shal_1516, JPD-1_1501, SL2-2_1608, ACS_DCE__8, Smul_1557, and N_1565). The architecture of the active site of PCE-to-TCE and PCE-to-*c*DCE dechlorinating RdhAs of *Sulfurospirillum* appears to be similar (26). Based on the structure of PceA in *S. multivorans* (27), one has to assume that the residues identified here are not directly involved in the formation of the enzyme’s active-site cavity. However, these residues are located in close vicinity to amino acids lining the substrate binding site and might influence the substrate specificity of the enzyme indirectly. Whether the residue substitution changed the substrate specificity could not be confirmed yet. Therefore, the development of efficient tools to edit the genetic contents of *Sulfurospirillum* will assist us in understanding these versatile microorganisms regarding organohalide respiration.

Despite physiological and evolutionary differences among the eight OHR *Sulfurospirillum* species strains, the second copies of RdhAs (e.g., cluster I) are highly conserved (i.e., >99% amino acid sequence identity) in all eight OHR *Sulfurospirillum* strains (14, 28). Generally, reductive dehalogenases are known to be phylogenetically diverse (29), and we did not expect to observe that the second copies of RdhAs with unknown functions were highly conserved in OHR *Sulfurospirillum* species strains distributed in geographically distinct locations. Despite the detection of the second copy of RdhA in the transcriptomic and proteomic studies on *S. multivorans* grown on PCE (14, 24), the function of the second copy of RdhA remains unclear. Hypothetically, the two genes encoding RdhAs (i.e., *pceA* and the second copy of the *rdhA* gene) were acquired horizontally before the speciation of the genus *Sulfurospirillum*, and more evolutionary pressures could be forced on the functional *pceA* gene than the second copy of the *rdhA* gene.

### Genome-inferred metabolic capacities and electron transport mechanism in *Sulfurospirillum*.

In addition to OHR, *Sulfurospirillum* species strains can conserve energy using a variety of electron acceptors (e.g., fumarate, dimethyl sulfoxide [DMSO], thiosulfate, arsenate, selenate, and nitrate) and electron donors (e.g., hydrogen, formate, hydrogen sulfide, lactate, and pyruvate) (4). For example, *S. halorespirans* strain PCE-M2 possesses a gene cluster encoding the SoxCDXYZAB proteins for thiosulfate oxidation. By comparison, other *Sulfurospirillum* species strains, including strain ACS_DCE_ and strain ACS_TCE_, have only two copies of the *phsA* gene encoding thiosulfate reductase. *Sulfurospirillum* species strains have been implicated in arsenate reduction with the potential of mobilizing arsenic in underground aquifers. While a single copy of the arsenate reductase gene *arsC* is present in the genomes of strains ACS_DCE_, ACS_TCE_, and JPD-1, other OHR *Sulfurospirillum* species strains harbor multiple copies of the *arsC* gene as well as arsenite oxidase genes *aioAB* (14). The ability to oxidize arsenite to arsenate at the presence of azurin protein as an electron acceptor was not observed in pure culture study, demonstrating that *aioAB* genes may not be functioning.

The electron transport chain of OHR can be generally classified into two categories: quinone dependent, represented by *Sulfurospirillum*, and quinone independent, represented by *Dehalococcoides* (12, 26, 30). OHR *Sulfurospirillum* species strains are quinone dependent and likely express the NapGH-like quinol dehydrogenases to transfer electrons from the menaquinone pool to PceA, similar to the function of NapGH quinol dehydrogenase in nitrate reduction (12, 24, 26). One of the possible electron sources for replenishing the electron pool is through oxidizing pyruvate via two different enzymes: pyruvate:ferredoxin/flavodoxin oxidoreductase (PFOR) and ubiquinone-dependent pyruvate dehydrogenase (PoxB) (14). The PFOR protein catalyzes the conversion of pyruvate to acetyl-CoA and carbon dioxide with simultaneous transfer of two electrons to ferredoxin or flavodoxin, while the PoxB enzyme could convert pyruvate into acetate and carbon dioxide accompanied by transferring the generated electrons directly to the menaquinone pool (31). The gene encoding PFOR has been found in all 12 *Sulfurospirillum* genomes; by comparison, the *poxB* genes are only present in the eight OHR *Sulfurospirillum* genomes, suggesting that *poxB* is probably related to the electron transfer chain of OHR. The inferred *poxB* products of OHR *Sulfurospirillum* species strains share 86.9% to 100% amino acid similarities to each other and are closely related to the ones identified in *Malaciobacter marinus* and gammaproteobacterial microorganisms (Fig. S8). The coexistence of both genes in a bacterial genome is uncommon, and the advantages for OHR *Sulfurospirillum* species strains possessing the *pfoR* and *poxB* genes simultaneously are not clear.

10.1128/msphere.00931-21.9 FIG S8 Phylogenetic analysis of PoxB from selected microorganisms. The PoxB homologs from *Sulfurospirillum* species strains were colored in blue, and the PoxB homologs from *Gammaproteobacteria* were colored in olive. The scale bar represents substitution per site. Download FIG S8, TIF file, 0.9 MB. Copyright © 2022 Yang et al. 2022 Yang et al. https://creativecommons.org/licenses/by/4.0/ This content is distributed under the terms of the Creative Commons Attribution 4.0 International license.

### Summary.

In this study, we performed phylogenetic, pangenomic, and evolutionary analyses on 12 complete *Sulfurospirillum* genomes, including two newly sequenced ones of strain ACS_DCE_ and strain ACS_TCE_ and proposed a new species, “*Candidatus* Sulfurospirillum acididehalogenans,” represented by strains ACS_TCE_, ACS_DCE_, and JPD-1. The relatively preserved region identified in all examined OHR *Sulfurospirillum* species strains, but not *S. multivorans* strains, suggested that they have a common ancestor that acquired the OHR capability recently and an additional insertion event(s) occurred in *S. multivorans* strains. Comparison between the PCE-to-TCE RdhAs and PCE-to-*c*DCE RdhAs identified the differences of four amino acid residues, but how these residue differences affect the substrate specificity of reductive dehalogenases remains unclear. The versatile metabolisms in *Sulfurospirillum* species strains ensure their great potentials in biotechnological applications, including the cleanup of soil and groundwater contaminated with a combination of chemicals (e.g., mixtures of arsenate, chlorinated ethenes, nitrate, and selenate). The ability to grow under unfavorable conditions (e.g., low pH) further emphasizes their roles and functions in specialized environmental settings. Nevertheless, some predictions on the metabolisms and capabilities of *Sulfurospirillum* were solely based on *in silico* genomic analysis, and experimental evidence is warranted in future studies. The plasmids harbored by strain ACS_TCE_ and strain ACS_DCE_ represent a promising tool for developing a robust gene editing tool that can advance the understanding of physiology and metabolism in the genus *Sulfurospirillum*.

## MATERIALS AND METHODS

### Sequence data set and phylogenetic analysis.

As of January 2022, 47 *Sulfurospirillum* assemblies are publicly available (https://www.ncbi.nlm.nih.gov/assembly/?term=Sulfurospirillum). CheckM (version 1.0.18) (32) assessment showed that the average completeness and contamination of 35 assemblies were 85.5% and 0.9%, respectively. Since the incompleteness and contamination of metagenome-assembled draft genomes (33) may affect the analysis of core, auxiliary, and singleton families, we only focused on the completed *Sulfurospirillum* genomes in this study. Among them, 12 completed *Sulfurospirillum* genomes, including the ones of strain ACS_TCE_ and strain ACS_DCE_, were selected for the following analyses (see Table S1 in the supplemental material and supplemental tables at https://doi.org/10.6084/m9.figshare.17014352.v1). Genome sequences of strain ACS_TCE_ and strain ACS_DCE_ were recently deposited and are publicly available in the NCBI genome database (21, 22). Phylogenetic analysis of *Sulfurospirillum* 16S rRNA gene sequences, which were retrieved from the Ribosomal Database Project (RDP) database, release 11 update 5 (34), was performed using PhyML with the general time-reversible (GTR) substitution model (35). Whole-genome-based taxonomy was analyzed using the Type (Strain) Genome Server (TYGS) (36) and Genome Taxonomy Database (GTDB), as described previously (37, 38). Briefly, RNAmmer v1.2 was applied to extract *Sulfurospirillum* 16S rRNA gene sequences (39), which were compared with those of available 10,997 type strains in the TYGS database for finding additional closely related type strains. Genomes were selected for pairwise comparisons using GBDP (Genome BLAST Distance Phylogeny) and accurate intergenomic distances (40), and the results were used to construct phylogenetic trees with a balanced minimum evolution via FASTME 2.1.4 (41). Species boundary was defined as 70% DNA-DNA hybridization (DDH). Average nucleotide identity (ANI) (e.g., ANIm [42], ANIb [43]) was calculated using JSpeciesWS (44) and orthoANI (45) to evaluate if two or more genomes can be classified into the same species. The ANI threshold for species boundary is defined as 95%.

### Molecular clock analysis.

MEGA X (46) for molecular evolutionary genetics analysis was applied to predict the divergence times using the RelTime method (47, 48) and the Tamura-Nei model (49). The 16S rRNA gene sequences were aligned with MUSCLE using the unweighted pair group method using average linkages algorithm and then analyzed for phylogeny reconstruction with the minimum evolution method or neighbor-joining method using Mega X. The nucleotide sequence alignment and phylogenetic tree were used as the input for RelTime-ML. The divergence times shown in Fig. S1 for several *Sulfurospirillum* species were predicted by TimeTree (50, 51) and were applied to set the divergence time calibration constraints by following the published approach (18, 52).

10.1128/msphere.00931-21.2 FIG S1 Phylogenetic tree and estimated divergent time of selected *Sulfurospirillum* species strains by TimeTree with all default settings (www.timetree.org). The predicted divergent times for labeled nodes were retrieved from the TimeTree database. Download FIG S1, TIF file, 0.7 MB. Copyright © 2022 Yang et al. 2022 Yang et al. https://creativecommons.org/licenses/by/4.0/ This content is distributed under the terms of the Creative Commons Attribution 4.0 International license.

### Whole-genome comparison and pangenome analysis.

*Sulfurospirillum* genomes were reannotated using the RAST (Rapid Annotation using Subsystem Technology) tool with default parameters (53, 54) and eggNOG-mapper (55–57) to ensure annotation conformity with formats and consistency across all genomes. Annotated coding sequences were verified by BLAST search (58) against NCBI nonredundant protein sequences and by UniProt database search (e.g., UniProtKB reference proteomes plus Swiss-Prot) (59). All 12 completed *Sulfurospirillum* genomes were used for pangenome analysis, which was constructed and performed by OrthoMCL (60) with default parameters using the KBase platform (61). The protein sequences of the key functions were retrieved and analyzed using KEGG (https://www.kegg.jp) (62, 63) and BRENDA (www.brenda-enzymes.org) (64) to identify the key pathways and modules among various *Sulfurospirillum* species strains.

### Analysis of functional sequences.

Protein sequences for building the phylogenetic inference tree were retrieved from the UniProt database (www.uniprot.com) (59). All protein sequences for building the phylogenetic trees of reductive dehalogenases are listed in the Text S1 (see also Data Set 1 at https://doi.org/10.6084/m9.figshare.17014145.v1) (65). The phylogenetic trees for reductive dehalogenases and transposases were built with Geneious software version 11.1.5 using the MUSCLE and FastTree or PhyML with default settings (Biomatters Inc., Newark, NJ, USA).

10.1128/msphere.00931-21.1 TEXT S1 Materials and methods of cultivation, metabolite detection, analytical methods, and data availability. Download Text S1, DOCX file, 0.02 MB. Copyright © 2022 Yang et al. 2022 Yang et al. https://creativecommons.org/licenses/by/4.0/ This content is distributed under the terms of the Creative Commons Attribution 4.0 International license.
