# Supplementary figures and images for: Comparative Genomic Analysis Reveals Preserved Features in Organohalide-Respiring Sulfurospirillum Strains
Source: mSphere. 2022 Feb 23;7(1):e00931-21. doi: 10.1128/msphere.00931-21 (PMC8865925; doi:10.1128/msphere.00931-21)

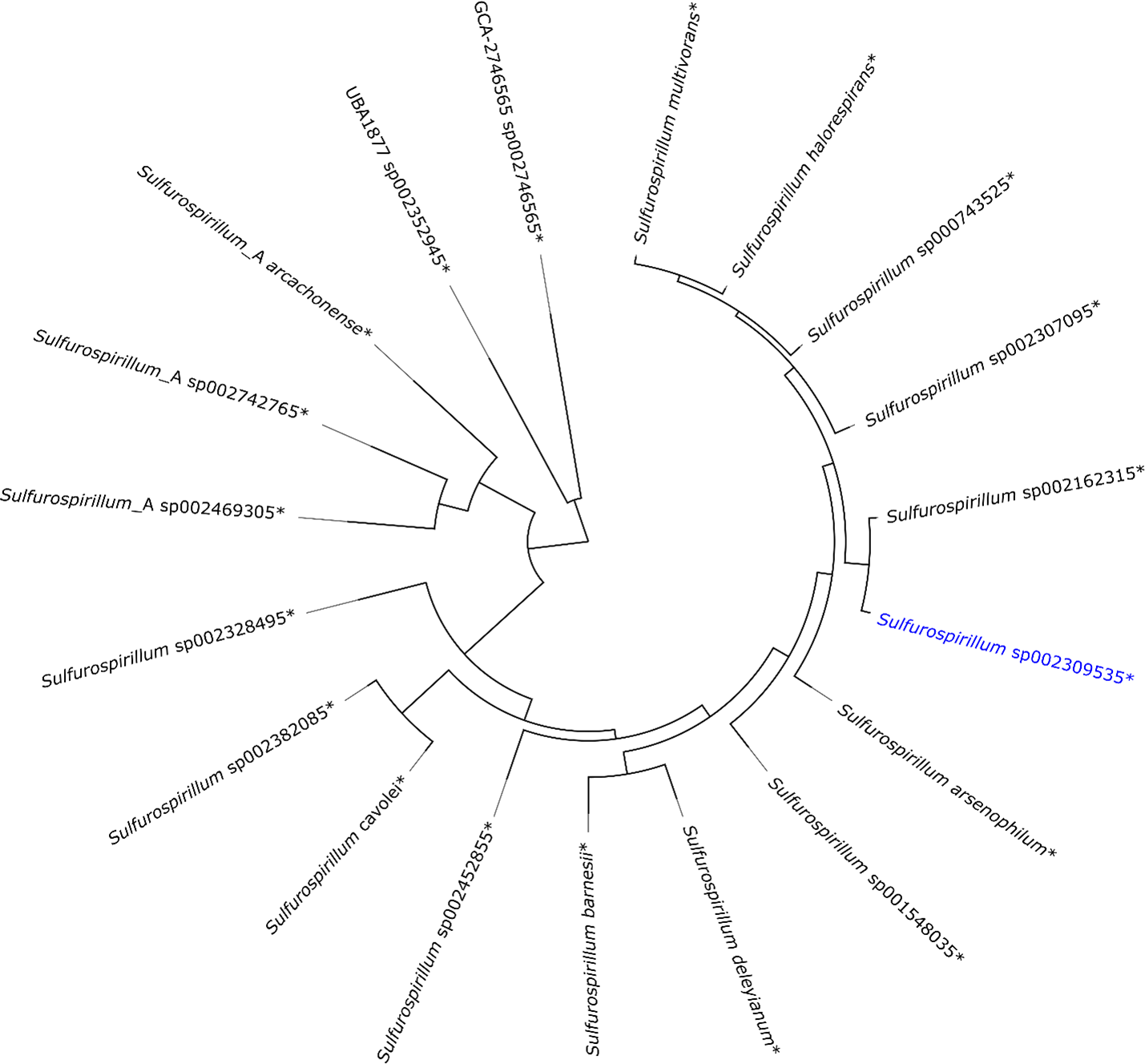

Supplement: FIG S2 [file msphere.00931-21-sf002.tif]

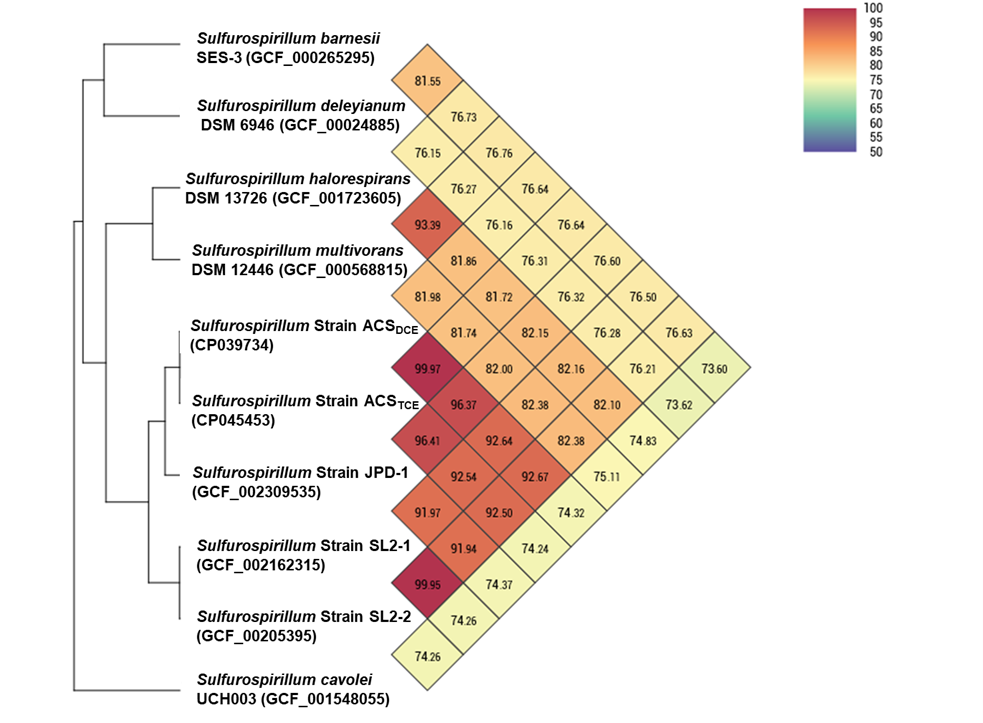

Supplement: FIG S3 [file msphere.00931-21-sf003.tif]

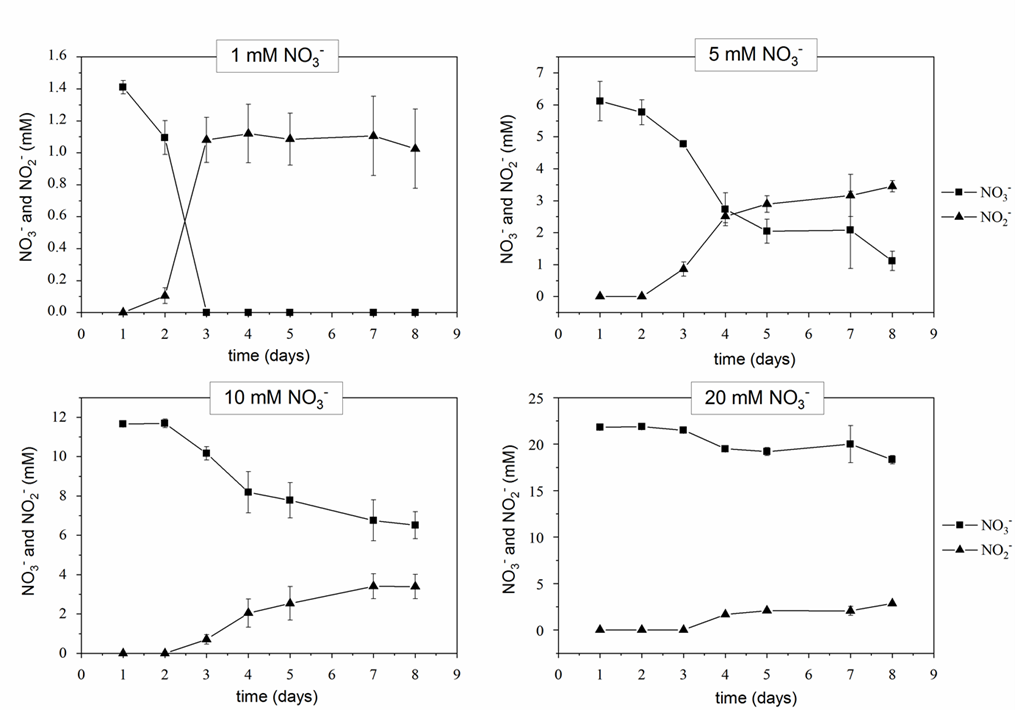

Supplement: FIG S4 [file msphere.00931-21-sf004.tif]

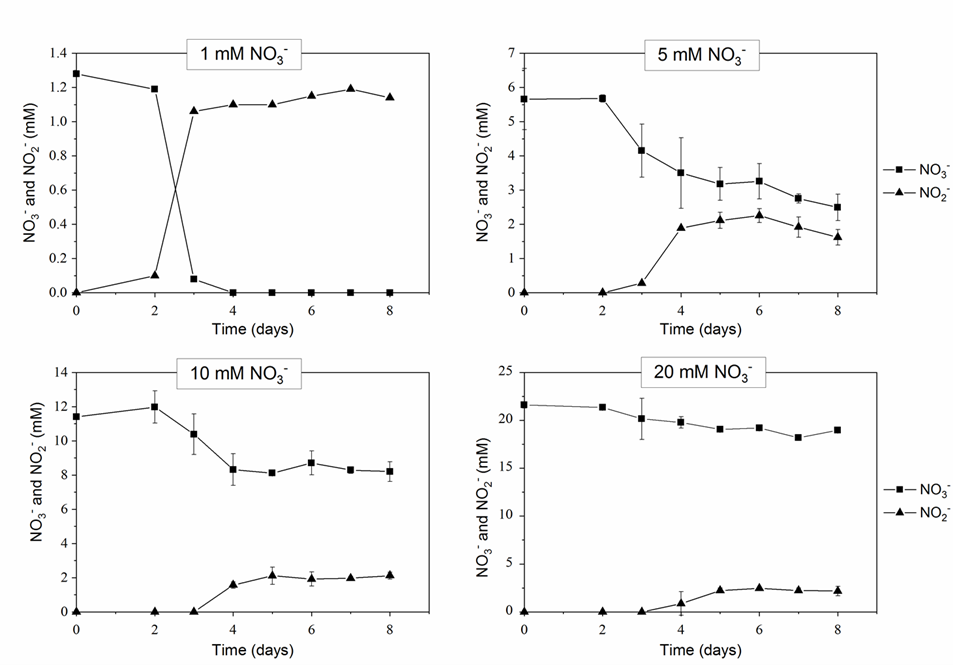

Supplement: FIG S5 [file msphere.00931-21-sf005.tif]

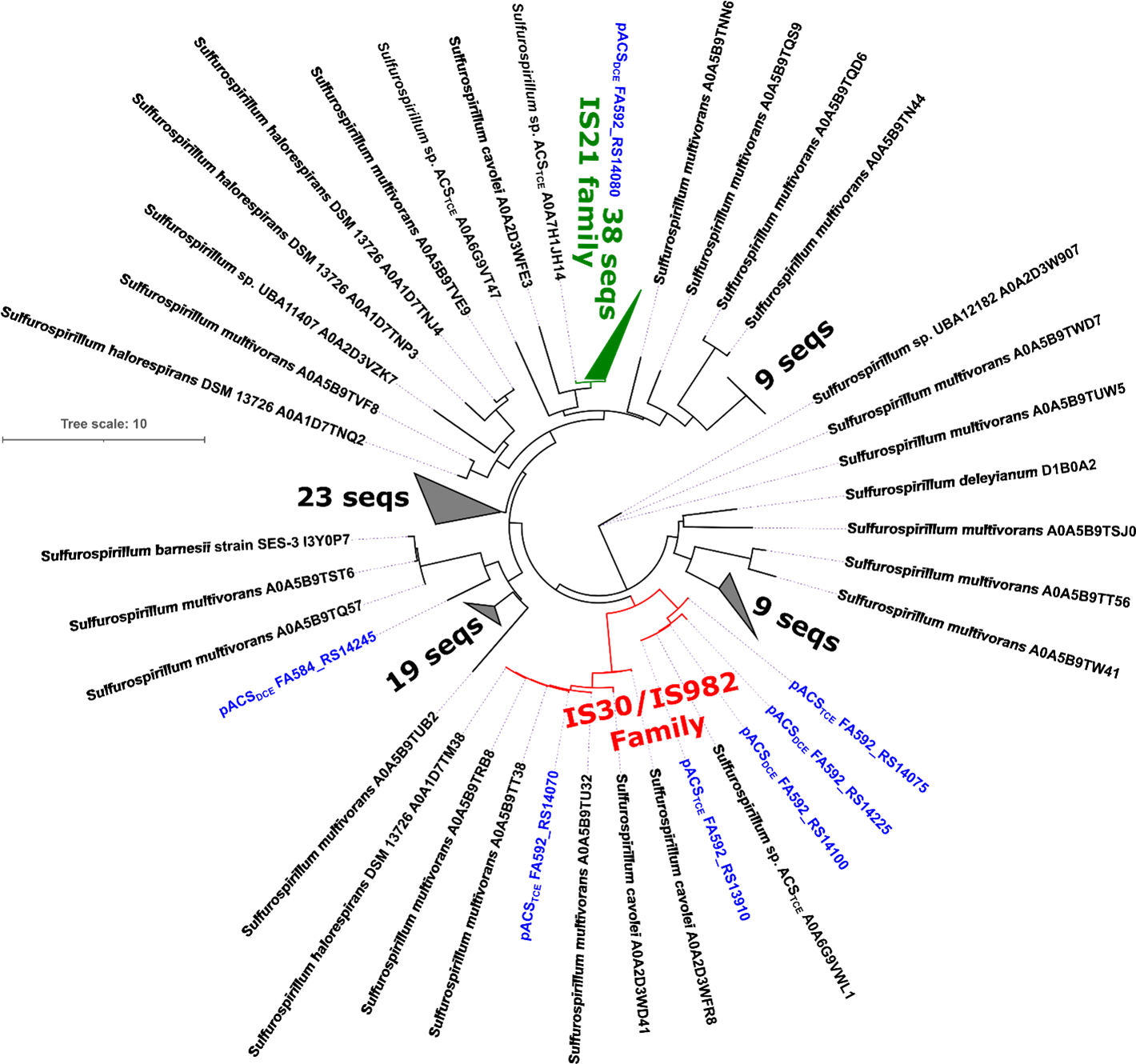

Supplement: FIG S6 [file msphere.00931-21-sf006.tif]

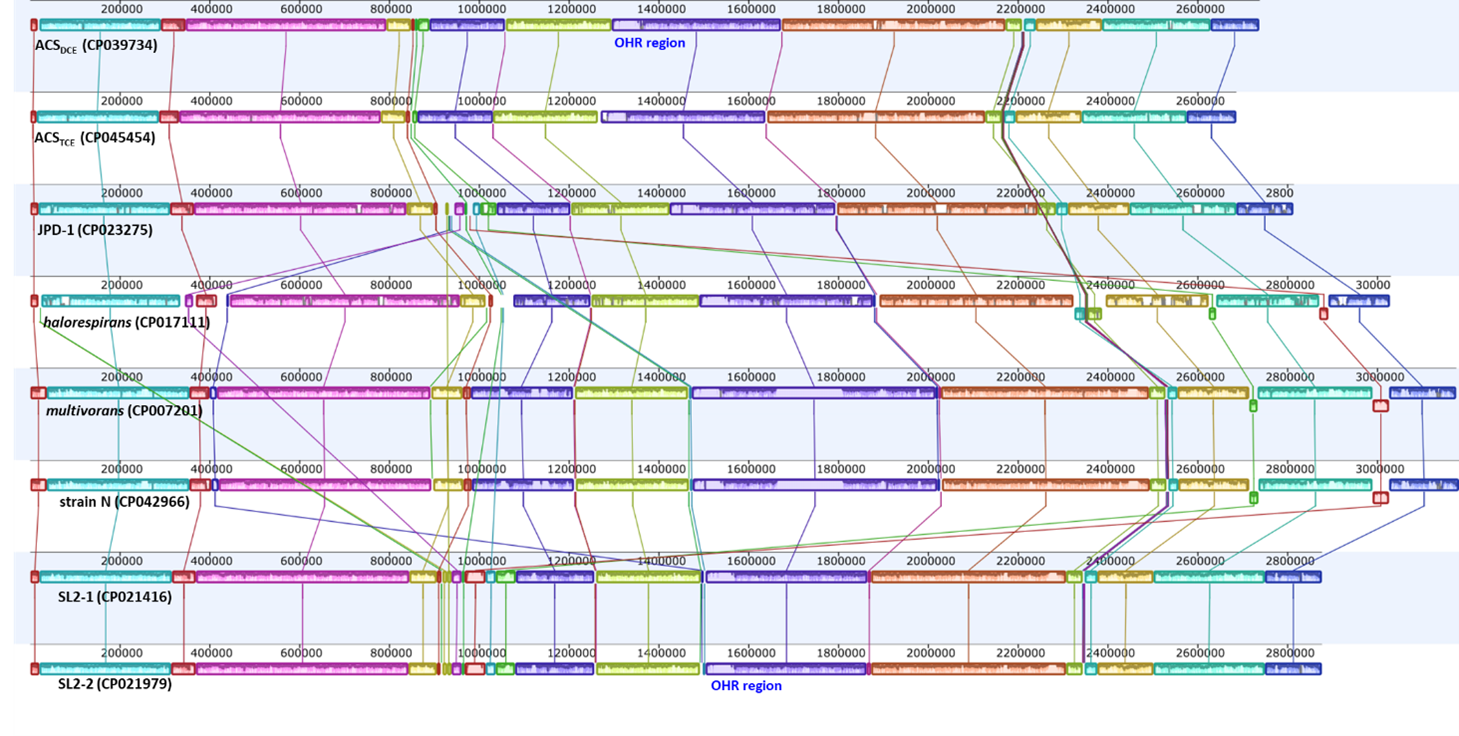

Supplement: FIG S7 [file msphere.00931-21-sf007.tif]

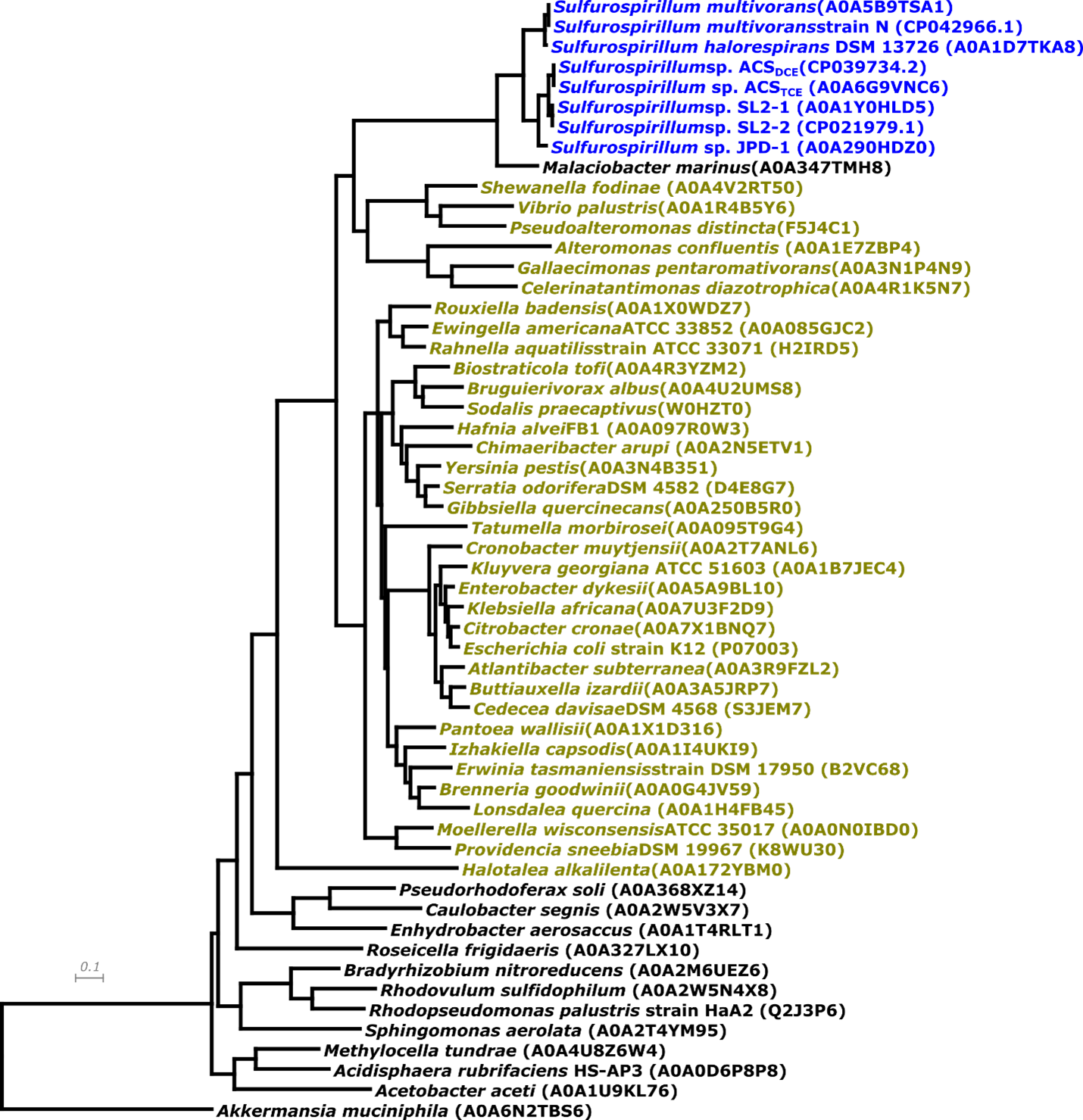

Supplement: FIG S8 [file msphere.00931-21-sf008.tif]

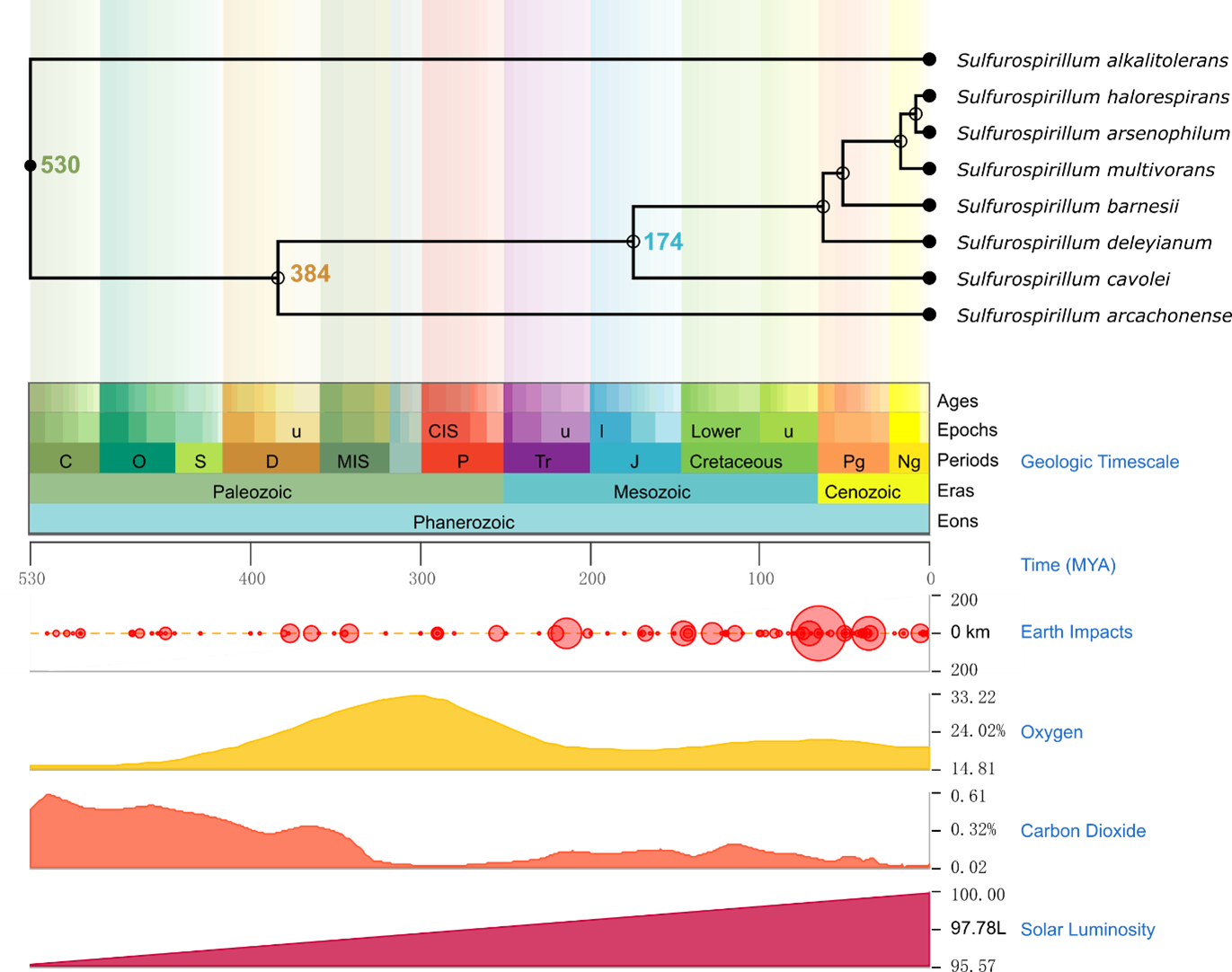

Supplement: FIG S1 [file msphere.00931-21-sf001.tif]
