# Supplementary material for: Comparative Genomic Analysis Reveals Preserved Features in Organohalide-Respiring Sulfurospirillum Strains
Source: mSphere. 2022 Feb 23;7(1):e00931-21. doi: 10.1128/msphere.00931-21 (PMC8865925; doi:10.1128/msphere.00931-21)
Supplement: TEXT S1 [file msphere.00931-21-s0001.docx]

Comparative Genomic Analysis Revealed Preserved Features in Organohalide-respiring *Sulfurospirillum* strains

Running Title: Differences between OHR- and non-OHR *Sulfurospirillum*

Yi Yang^1*^, Torsten Schubert^3^, Yan Lv^1,3^, Xiuying Li^1^, Jun Yan^1*^

^1^Key Laboratory of Pollution Ecology and Environmental Engineering, Institute of Applied Ecology, Chinese Academy of Sciences; Shenyang, Liaoning, China, 110016; ^2^Research Group Anaerobic Microbiology, Institute of Microbiology, Friedrich Schiller University Jena, Jena, Germany, 07743; ^3^University of Chinese Academy of Sciences, Beijing, China, 100049.

* Corresponding authors

Jun Yan

Key Laboratory of Pollution Ecology and Environmental Engineering

Institute of Applied Ecology, Chinese Academy of Sciences

Shenyang, Liaoning 110016, China

Phone: +86-24-83970426

E-mail: junyan@iae.ac.cn

Yi Yang

Key Laboratory of Pollution Ecology and Environmental Engineering

Institute of Applied Ecology, Chinese Academy of Sciences

Shenyang, Liaoning 110016, China

Phone: +86-24-83970426

E-mail: [yangyi@iae.ac.cn](mailto:yangyi@iae.ac.cn)

**Supplementary Materials**

**Cultures and growth conditions.** *Sulfurospirillum* sp. strain ACS_DCE_ and strain ACS_TCE_ cultures were grown in 160 mL serum bottles containing a N_2_/CO_2_ (80/20, v/v) headspace and 100 mL bicarbonate buffered (30 mM, pH 7.2) mineral salt medium amended with nitrate (1, 5, 10 or 20 mM) (1). Formate (10 mM) and acetate (5 mM) were provided as electron donor and carbon sources, respectively. Each bottle received a 3% (v/v) inoculum from a strain ACS_DCE_ or strain ACS_TCE_ culture previously grown with PCE as the electron acceptor (1). All bottles were incubated statically at 30 ^o^C in the dark.

**Cultivations and metabolite detection.** Anhydrous sodium acetate (≧99.0% ), sodium nitrate (≧99.0%), sodium nitrite(≧99.0%), N_2_O(≧99.0%), ammonium chloride (≧99.5%), PCE (99.0%), TCE(≧99.5%) and *c*DCE (>97.0%) were all purchased from Sigma-Aldrich Chemicals (St. Louis, MO, USA). *Sulfurospirillum* strains ACS_DCE_ and ACS_TCE_ were grown in the 100 ml sodium sulfide-free and anoxic defined mineral salts medium prepared following the published protocol (2). Acetate (5 mM), formate (10 mM), and PCE (50 μM) were added as the carbon source, electron donor, and electron acceptor, respectively. Duplicate cultures were incubated at 30°C in the dark without shaking. Negative controls were set up by autoclaving the inoculated vessels. *Sulfurospirillum* strains ACS_DCE_ and ACS_TCE_ were tested for nitrate reduction by cultivation with formate (10 mM) as the electron donor and nitrate (1, 5, 10 or 20 mM) as the sole electron acceptor. Nitrate and nitrite were quantified by Agilent 1260 high-pressure liquid chromatography (HPLC) (Agilent Technologies, Santa Clara, USA) equipped with a C18 column (250 × 4mm, 5μM, Thermo Fisher, Waltham, MA, USA). The mobile phase was 30 mM phosphate buffer (pH= 2.8)/acetonitrile (95/5, v/v) at a flow rate of 0.6 mL/min. The column temperature was set at 35 °C. Absorbances of eluted compounds were monitored at 215 nm. Liquid samples (0.4–0.5 mL) were withdrawn from serum bottles by using a 1 mL syringe (Chifeng Tianbo Medical Equipment Co., Ltd, China) and were filtered through the 0.22 um filters (Tianjin Jinteng Experiment Equipment Co., Ltd, China) at 1-day intervals. The samples were stored at 4°C before analysis. PCE, TCE and *c*DCE were measured as described previously (1, 3).

**Analytical methods.** Supernatant samples were prepared by passing 0.5 mL culture suspensions through 0.22-µm sterile syringe filters and stored at 4°C. Nitrate and nitrite were analyzed using an Agilent 1260 high performance liquid chromatography (HPLC) equipped with a C18 column (250 mm length × 4 mm diameter, 5 μm particle size; Thermo Fisher, Waltham, MA, USA) operated at 35 °C. Samples were separated with isocratic elution of 30 mM phosphate buffer (pH 2.8) and 100% acetonitrile (95/5, v/v) at a flow rate of 0.6 mL/min and detected with a diode-array-detector (DAD) set at 215 nm.

**Data availability.** Supplementary materials and data were stored in the figshare platform with links https://figshare.com/articles/dataset/Supplemental_Material_Table_S1-S9/17014352 and https://figshare.com/articles/dataset/Supplementary_Dataset_1/17014145.

**References**

1. Yang Y, Capiro NL, Marcet TF, Yan J, Pennell KD, Löffler FE. 2017. Organohalide respiration with chlorinated ethenes under low pH conditions. Environ Sci Technol 51:8579-8588.

2. Löffler FE, Sanford RA, Ritalahti KM. 2005. Enrichment, cultivation, and detection of reductively dechlorinating bacteria. Methods Enzymol 397:77-111.

3. Yang Y, Higgins SA, Yan J, Simsir B, Chourey K, Iyer R, Hettich RL, Baldwin B, Ogles DM, Löffler FE. 2017. Grape pomace compost harbors organohalide-respiring *Dehalogenimonas* species with novel reductive dehalogenase genes. ISME J 11:2767-2780.
